# Polymyalgia rheumatica presenting as sternoclavicular arthritis: a case report

**DOI:** 10.1186/s13256-022-03661-8

**Published:** 2022-12-16

**Authors:** Liyana Arachchige Dona Thulini Madubashini, Jayawardane Pathiranage Roneesha Lakmali, Nilanka Perera

**Affiliations:** 1 Professorial Medicine Unit, Colombo South Teaching Hospital, Kalubowila, Nugegoda, Sri Lanka; 2 Department of Medicine, Faculty of Medical Sciences, University of Sri Jayewardenepura, Nugegoda, Sri Lanka

**Keywords:** Case report, Sternoclavicular arthritis, Polymyalgia rheumatica, Giant cell arteritis, Ureteric stricture, Vasculitis

## Abstract

**Background:**

Polymyalgia rheumatica and giant cell arteritis are systemic inflammatory conditions of the elderly. Polymyalgia rheumatica classically presents as a bilateral proximal muscle pain and stiffness syndrome. Biceps tenosynovitis is the commonest pathology in polymyalgia rheumatica. However according to literature, erosive sternoclavicular arthritis is a rare association of polymyalgia rheumatica. Giant cell arteritis is an inflammatory granulomatous arteritis predominantly involving large cerebral arteries. Thus, its classic clinical presentation includes severe headache with scalp tenderness, jaw claudication, and sudden painless loss of vision. Urological manifestations (prostatic vasculitis and epididymo-orchitis) were seldom reported in giant cell arteritis.

**Case presentation:**

A 53-year-old Sinhalese man presented with progressive right-sided shoulder joint pain and neck pain associated with constitutional symptoms and episodic generalized headache. Examination revealed restricted movements of the right shoulder joint with nontender pulsatile bilateral temporal arteries. Blood testing showed elevated erythrocyte sedimentation rate and C-reactive protein. Color Doppler ultrasound of the superficial temporal artery revealed “halo sign.” The temporal artery showed infiltration of mononuclear cells in the arterial media and adventitia. Computed tomography revealed right sternoclavicular arthritis with incidental finding of ureteric stricture. The patient was treated with high-dose oral prednisolone, and good clinical and biochemical response was observed during follow-up.

**Conclusion:**

Polymyalgia rheumatica–giant cell arteritis may rarely present as erosive sternoclavicular arthritis as the initial manifestation, mimicking many rheumatological conditions. Urological involvement such as ureteric strictures may be rare associations of primary systemic vasculitis. A high degree of suspicion combined with targeted investigations would allow early identification the polymyalgia rheumatica–giant cell arteritis syndrome in the presence of atypical manifestations, leading to improved patient outcomes.

## Introduction

Polymyalgia rheumatica (PMR) is an inflammatory rheumatic condition clinically characterized by bilateral symmetrical shoulder, hip, and neck pain with morning stiffness lasting more than 45 minutes with unknown etiology [1]. It is a disease of people over the age of 50 years. It is known to be associated with giant cell arteritis (GCA), and PMR is two to three times commoner than GCA. Though the term “polymyalgia rheumatica” implies a disease of muscle, muscle biopsy is normal in this condition. In fact, periarticular (bursae and tendons) and articular structures are involved in PMR. While bilateral subdeltoid/subacromial bursitis is the hallmark of PMR, other manifestations such as bicep tenosynovitis, glenohumeral synovitis, cervical and lumbar interspinous bursitis, iliopectineal and iliopsoas bursitis, hip synovitis, and hamstring tendinitis occur.

Herein, we report a case of unilateral sternoclavicular arthritis in a patient diagnosed with PMR associated with GCA.

## Case presentation

**Fig. 1:**
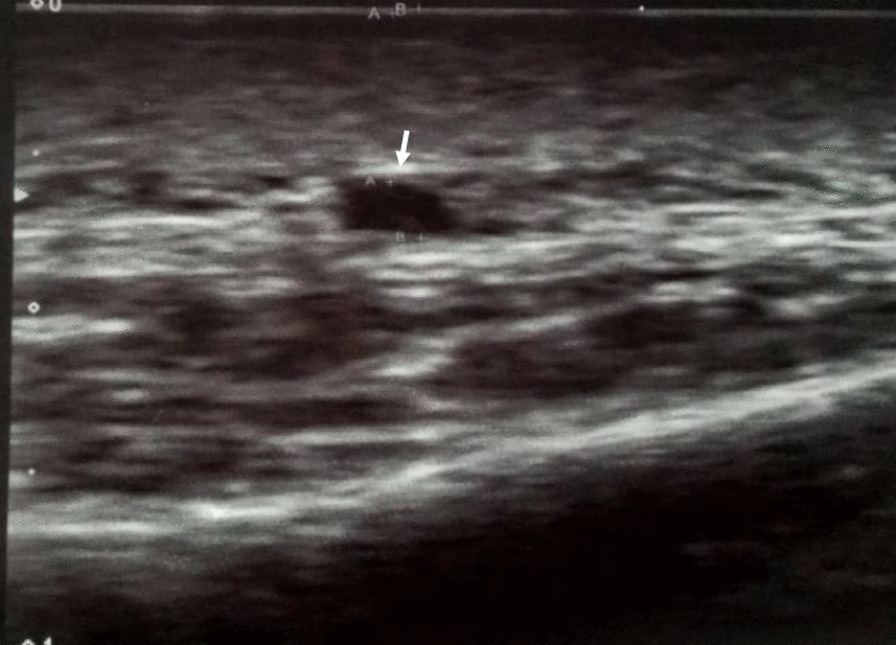
Cross-sectional Doppler ultrasound view of right temporal artery. Arrow is pointing to hypoechogenic halo sign

A 53-year-old Sinhalese, known patient with long-standing type 2 diabetes mellitus and hypertension was admitted with 1-week history of right-sided shoulder joint pain and neck pain with early morning stiffness for 1-hour duration leading to limitations in activities of daily living. On further inquiry, there was no joint pain involving the left shoulder, bilateral hip, and other small or large joints. He also complained of loss of appetite, lethargy, and generalized malaise. He reported history of intermittent generalized headache without blurring of vision or jaw claudication with duration of last 2 weeks. There was no history suggestive of ureteric colic or history of urological surgeries and radiation therapy. There was no family history of autoimmune or connective tissue diseases. On examination, he was pale and afebrile and there was no cervical lymphadenopathy or rashes. There were restricted movements of the right shoulder joint and neck with normal examination of other joints. Bilateral temporal arteries were nontender and pulsatile. Respiratory, cardiovascular system, and abdominal examinations were unremarkable (Fig. 1; Table 1).

**Table 1:** Laboratory investigations in the patient during evaluation

Investigation	Result	Reference range
White blood cells (× 10^9^/L)	13.9	4–10
Hemoglobin (g/dL)	10.2	12–16
Platelets (× 10^9^/L)	230	150–400
Erythrocyte sedimentation rate	85 mm /1st hour	
C-reactive protein (mg/dL)	73	< 5
Alkaline phosphatase (U/L)	581.2	30–120
Gamma-glutamyl transferase (U/L)	636.8	< 55
Urea (mmol/L)	8.9	2.1–8.5
Creatinine (µmol/L)	167	74–110
Sodium (mmol/L)	134.5	136–146
Potassium (mmol/L)	3.8	3.5–5.1
Calcium (mmol/L)	2.43	2.2–2.7
Creatine phosphokinase (U/L)	118	55–170
9 AM cortisol (nmol/L)	485	101–535
Aspartate transaminase (U/L)	22.2	< 50
Alanine transaminase (U/L)	32.8	< 50
Total protein (g/L)	68.22	66–83
Albumin (g/L)	26.7	35–52
Globulin (g/L)	41.52	25–35
Total bilirubin (µmol/L)	20.3	5.0–21.0
Rheumatoid factor	Negative	
Urine culture	No growth	
Blood culture	No growth	
Sputum for acid-fast bacilli	Negative	
Mantoux test	Negative	
Shoulder joint and cervical spine radiographs	Normal	
Chest radiograph	Normal	
Ultrasound of abdomen	Left-side hydronephrosis and hydroureter (grade 2)	
Noncontrast computed tomography of kidney–ureter–bladder	Left-side moderate hydronephrosis and hydroureter owing to distal ureteric stricture; no evidence of urolithiasis	
Contrast tomography of chest and abdomen	Right sternoclavicular joint arthropathic changes Gall bladder normal in size with no evidence of calculi within; biliary tree not dilated; liver normal, and rest of the study unremarkable	
Serum protein electrophoresis	Mild hypoalbuminemia, polyclonal hypergammaglobulinemia Features suggestive of acute on chronic inflammation	
Ultrasound Doppler of temporal artery	Thickening of right-side temporal artery with halo sign with normal left-side temporal artery suggestive of right-side temporal arteritis	
Biopsy of right-side temporal artery	Infiltration of mononuclear cells in the arterial media and adventitia	

Computed tomography scan revealed hypodense content in the right sternoclavicular joint with adjacent small focal bone erosions likely due to arthropathic changes without evidence of bursitis or tendinitis suggestive of sternoclavicular arthropathy.

A final diagnosis of PMR presenting as right sternoclavicular arthritis with GCA was made. He was started on high-dose oral prednisolone, with good clinical and biochemical improvement.

## Discussion

PMR is the commonest inflammatory rheumatic disease in the elderly, classically manifesting as proximal pain and stiffness involving the shoulder, hip, and neck [1]. It has been found in studies that 21% of patients with PMR had coexisting GCA [2].

Biceps tenosynovitis is the commonest pathology identified by musculoskeletal ultrasound scan in patients with PMR, whereas other manifestations include subdeltoid bursitis, glenohumeral synovitis, and less frequently, hip involvement [3]. Sternoclavicular arthropathy is a rare association of PMR. According to literature, sternoclavicular arthritis was noted in 15% of the patients in one study [4]. Though the occurrence of erosive arthropathy is controversial in PMR, there is evidence that erosions of pubic symphysis, sacroiliac, and sternoclavicular joints occur, especially in patients with symptoms lasting more than 6 months [5].

PMR is diagnosed based on clinical presentation and supportive laboratory findings. Hence, there is no pathognomonic test or diagnostic criteria. The American College of Rheumatology and European Alliance of Associations for Rheumatology have proposed classification criteria for PMR, which should not be used as diagnostic criteria [6].

PMR has a good prognosis if diagnosis and treatment are prompt.

Though PMR classically causes bilateral symmetrical limb girdle pain, our patient presented with unilateral shoulder joint pain with neck pain lasting more than 2 weeks, with other supportive evidence such as early morning stiffness, age more than 50 years, and high inflammatory markers, viz. gamma-glutamyl transferase and alkaline phosphatase, in the presence of a negative rheumatoid factor. Computed tomography revealed erosive sternoclavicular arthritis, which is a rare manifestation of PMR.

At presentation, he had coexisting GCA, as proven by temporal artery ultrasound and biopsy, though the symptoms were not classic. A high degree of suspicion led to prompt diagnosis and treatment of PMR-GCA in this patient. Cholestatic hepatic dysfunction is common in patients with PMR but disappears following glucocorticoid treatment [7]. It indicates a high risk of having overlapping GCA. In our patient, we observed elevated alkaline phosphatase and gamma glutamyl transferase with normal bilirubin and aminotransferase levels, which is commonly reported in published literature. GCA is a large vessel vasculitis predominantly affecting aorta and its branches. Urological manifestations are seldom reported with GCA, including prostatic vasculitis and epididymo-orchitis. Urethral involvement was more commonly seen in polyarteritis nodosa and Henoch–Schonlein purpura, where the majority of cases resolved with systemic glucocorticoid treatment [8]. In our case, we recognized an incidental finding of hydronephrosis and hydroureter due to distal ureteric stricture.

The patient was started on high-dose systemic glucocorticoids with an initial dose of 60 mg of prednisolone, gradually tapered according to clinical and biochemical response. Good clinical and biochemical response was observed with treatment, and he was monitored for side effects of glucocorticoids and recurrence of symptoms in the medical clinic.

## Conclusion

Unilateral erosive sternoclavicular arthritis and ureteric strictures are both rare presentations of PMR and GCA, respectively. Such atypical manifestations mimic many rheumatological conditions, leading to a delay in diagnosis. Knowledge of such rare manifestations would ensure prompt diagnosis and treatment, which would significantly improve the outcome from PMR-GCA.

## Data Availability

All data generated or analysed during this study are included in this published article and its supplementary information files.

## Abbreviations

GCA: Giant cell arteritis
PMR: Polymyalgia rheumatica

## References

[1] Milchert M, Brzosko M. Diagnosis of polymyalgia rheumatica usually means a favourable outcome for your patient [Internet]. Indian J Med Res. 2017 [cited 2022Jun5]. Available from: https://www.ncbi.nlm.nih.gov/pmc/articles/PMC5644293/. 10.4103/ijmr.IJMR_298_17 PMC5644293 28948949

[2] Sharma A, Mohammad AJ, Turesson C. Incidence and prevalence of giant cell arteritis and polymyalgia rheumatica: a systematic literature review [Internet]. Semin Arthritis Rheum. 2020 [cited 2022Jun5]. https://www.sciencedirect.com/science/article/pii/S0049017220302043#:~:text=Around%2040%25%20to%2060%25%20of,of%20selection%20of%20severe%20cases. 10.1016/j.semarthrit.2020.07.005 32911281

[3] Weigand S, Ehrenstein B, Fleck M, Hartung W. Joint involvement in patients with early polymyalgia rheumatica using high-resolution ultrasound and its contribution to the EULAR/ACR 2012 classification criteria for polymyalgia rheumatica [Internet]. J Rheumatol. 2014;41:730. 10.3899/jrheum.130946 24584915

[4] MB; MLDS. Skeletal manifestations of polymyalgia rheumatica [Internet]. JAMA. U.S. National Library of Medicine; [cited 2022Jun5]. Available from: https://pubmed.ncbi.nlm.nih.gov/660807/.

[5] Paice EW, Wright FW, Hill AG. Sternoclavicular erosions in polymyalgia rheumatica. Ann Rheum Dis. 1983;42(4):379–83. 10.1136/ard.42.4.379 PMC1001246 6882033

[6] UpToDate. [cited 2022Jun5]. Available from: https://www.uptodate.com/contents/clinical-manifestations-and-diagnosis-of-polymyalgia-rheumatica.

[7] Hysa E, Castagna A, Manzo C. Liver involvement in polymyalgia rheumatica and giant cell arteritis. Reumatologia [Internet]. 2020;58(6):444–5. 10.5114/reum.2020.101580. PMC7792548 33456088

[8] Peracha J, Morgan MD. Urological manifestations and treatment of the primary systemic vasculitides [Internet]. World J Clin Urol. 2015;4:5.

